# Analysis of indoor radon concentration levels and trends in China

**DOI:** 10.3389/fpubh.2025.1524179

**Published:** 2025-02-04

**Authors:** Bowei Ding, Yunyun Wu, Yanchao Song, Changsong Hou, Bing Shang

**Affiliations:** Key Laboratory of Radiological Protection and Nuclear Emergency, China CDC, National Institute for Radiological Protection Chinese Center for Disease Control and Prevention, Beijing, China

**Keywords:** China, radon, indoor, database, regional analyses

## Abstract

A systematic review of publicly available papers on indoor radon data from 1980 to 2023 was conducted to provide a preliminary understanding of indoor radon concentration levels and trends in China. Keywords were used to collect literature on indoor radon surveys in China during the periods of before 2000, 2000–2010 and after 2010 in the CNKI, WANFANG, VIP and PubMed databases. This paper also collected indoor radon concentration data from WHO, UNSCEAR publications and PubMed databases for other countries. A total of 37,886 indoor radon concentration data points were collected in China, covering 31 provinces. The results showed that the weighted and arithmetic mean radon concentrations in China were 29.4 Bq/m^3^ and 33.2 Bq/m^3^ (*n* = 17,940) before 2000, 44.7 Bq/m^3^ and 43.3 Bq/m^3^ (*n* = 10,692) in 2000–2010, 57.6 Bq/m^3^ and 60.8 Bq/m^3^(*n* = 9,254) after 2010, respectively. It indicated an increasing trend in indoor radon concentrations in China. The differences in mean indoor radon concentrations across time periods were significant (*p* < 0.001). In the regional analysis, the differences in indoor radon concentrations between different administrative geographic regions for each time period were significant (*p* < 0.05). Furthermore, the differences in indoor radon concentrations among climatic areas were significant for the periods 2000–2010 and after 2010 (*p* < 0.05). Additionally, this paper collected indoor radon data from 63 countries worldwide. The mean radon concentrations across the three periods—before 2000, 2000–2010 and after 2010—were 56.5 Bq/m^3^, 67.9 Bq/m^3^ and 81 Bq/m^3^, respectively. Meanwhile, a comparison of indoor radon concentration was made before and after 2000 among 26 countries, of which 16 countries showed an increasing trend. So, it can be seen the increase in indoor radon concentration in China is not an isolated phenomenon, and the issue of indoor radon pollution still requires further attention.

## 1 Introduction

Radon is a radioactive gas that is widely distributed in nature. It is colorless, odorless and pervasive, and it constitutes the primary component of natural radiation exposure (1). There are 27 known isotopes of radon, of which ^219^Rn, ^220^Rn and ^222^Rn are three natural radioisotopes. This paper focuses on ^222^Rn. ^222^Rn arises from the natural radioactive uranium decay system existing in the rocks and soil of the earth, and ^238^U generates ^226^Ra after a series of decays with a half-life of 1,602 years, which is the direct parent of ^222^Rn. Given the prevalence of radioactive elements such as uranium and radium in soil, rock and groundwater, it is inevitable that radon will be released from these medium and enter the surface and indoor environment through advection and diffusion (2). The primary sources of indoor radon are the house foundation and surrounding soil, building materials, outdoor air, domestic water, natural gas, and household fuels (2, 3). Among these sources, building materials represent the primary source of indoor radon in multi-storey or high-rise buildings. Epidemiological studies (4–11) have demonstrated that radon is the second leading cause of lung cancer in the general population, after smoking. These studies provide compelling evidence of an association between indoor radon exposure and lung cancer, even at relatively low radon levels in common dwellings (12). Therefore, indoor radon is one of the most significant air pollutants as people spend about 80% of their lives indoors (13, 14).

In order to mitigate the adverse effects of radon on human health and to develop effective risk management strategies, numerous countries have conducted comprehensive nationwide surveys on indoor radon concentrations and established indoor radon control standards (15–17). Since the 1980s, China has conducted successive national or regional surveys of indoor radon concentrations. From 1984 to 1990, the survey organized by the Ministry of Health (18) covering 26 provinces and cities with a total of 10,811 data points showed that the average indoor radon concentration was 22.5 Bq/m^3^. At the same time, a survey administered by the Ministry of Environmental Protection (19), covering 21 provinces and cities with a total of 1,610 data points, reported indoor radon concentration in China was 20.2 Bq/m^3^. From 2001 to 2004, the National Institute for Radiological Protection of the Chinese Center for Disease Control and Prevention (NIRP, CCDC) (20) conducted a survey on indoor radon concentrations in typical areas of China, including 18 provinces and cities with a total of 2,117 data points, and the results demonstrated that the average indoor radon concentration in China was 44.1 Bq/m^3^ in China. The increasing trend of indoor radon concentrations in China has raised widespread concern among researchers. Subsequently, from 2006 to 2010, two regional surveys on indoor radon concentration were conducted by the China National Nuclear Corporation and the Ministry of Housing and Urban–Rural Development (21, 22) respectively, with reported indoor radon concentrations of 32.6 Bq/m^3^ and 34.9 Bq/m^3^, respectively. However, after 2010, no national surveys of indoor radon concentrations were conducted in China, so it is necessary to conduct a comprehensive summary and comparison of national indoor radon surveys.

This paper provided a summary of indoor radon concentration survey data in China across three time periods: before 2000, 2000–2010, and after 2010. The analysis encompassed the levels and trends of indoor radon concentration across diverse administrative regions and building climatic areas. Additionally, the study made a comparative analysis of indoor radon concentration with other countries across different periods. The results of the analyses would provide a scientific basis to formulate building and indoor air quality standards. Simultaneously, it also could raise public awareness of indoor radon and enable the public to take corresponding protective measures, such as enhancing ventilation and using radon-resistant materials, to reduce the risk of lung cancer.

## 2 Methodology

**Figure 1:**
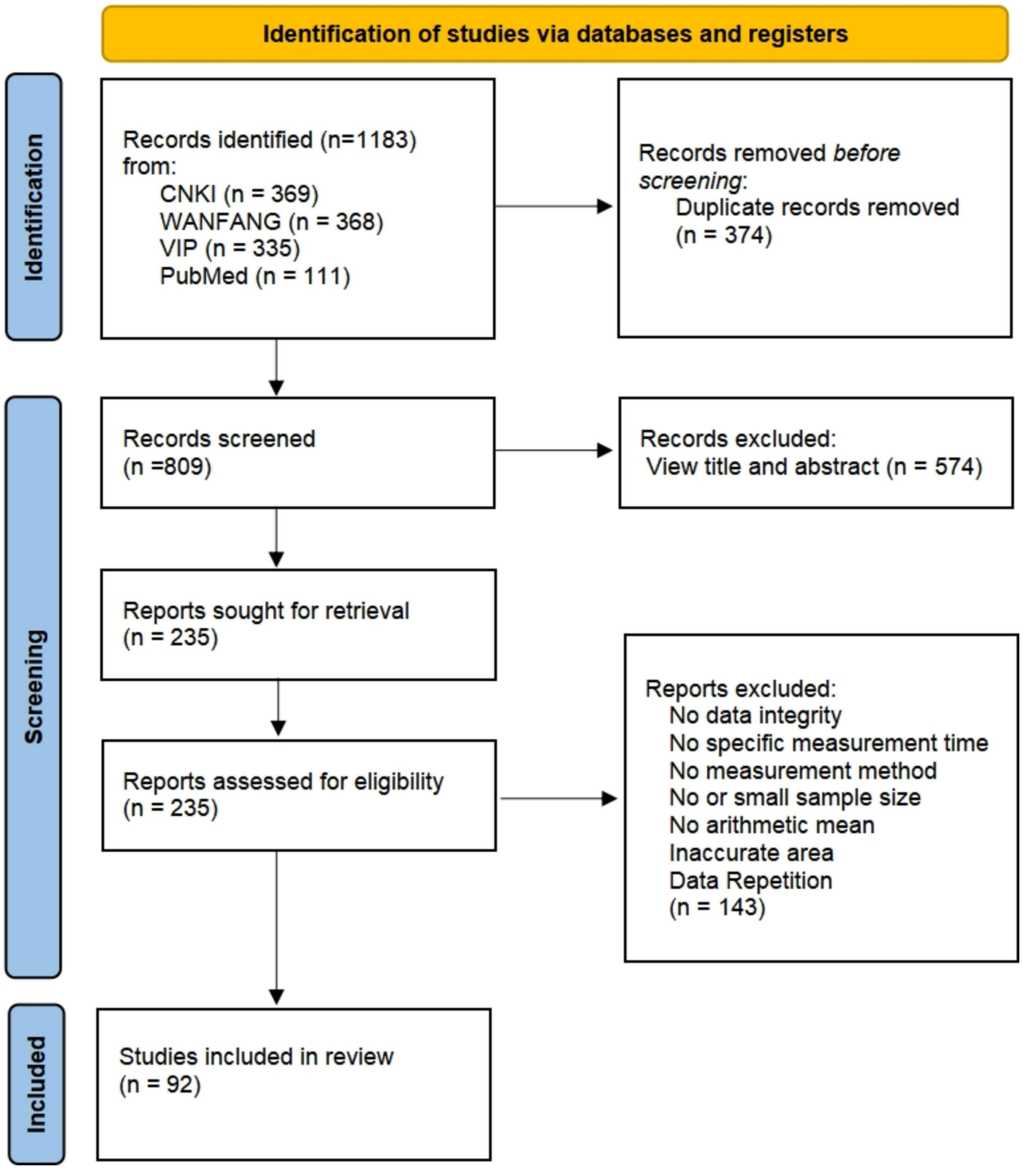
The literature search of indoor radon concentration data in China.

The literature on indoor radon concentrations in China was collected in three representative periods: before 2000, 2000–2010, and after 2010. It was done through the literature search platforms CNKI, WANFANG, VIP and PubMed, and the open literature was searched using the terms “radon,” “indoor,” “residential,” “concentration,” and “survey,” as shown in Figure 1. In addition to searching literature on nationwide indoor radon concentrations in other countries, publications from the World Health Organization (WHO) and the United Nations Scientific Committee on the Effects of Atomic Radiation (UNSCEAR) were also examined for national surveys on indoor radon concentrations. A preliminary screening was conducted based on the time and location of the survey, sample size, measurement method, and radon concentration and its statistical description (arithmetic mean, maximum and minimum values) of the collected literature in order to form an initial database of indoor radon concentration covering the world.

In the screening of literature containing indoor radon data, only papers presenting arithmetic means were included. When calculating the mean value for each province, the weighted arithmetic mean based on sample size was used if multiple surveys were available, to determine the average indoor radon concentration for that province. Similarly, when calculating the indoor radon concentration on a national scale, the sample size and mean value of each province were used to calculate the sample-weighted mean value. In contrast to the population-weighted calculation of the global mean radon concentration presented in the UNSCEAR report, this study employed the arithmetic mean as the global mean radon concentration, based on the collection of indoor radon concentration data from many countries worldwide in this study. In the comparison between China and other countries, the arithmetic mean of indoor radon concentration in China was calculated and then compared with the arithmetic means of other countries.

In order to evaluate the correlation between indoor radon concentrations and a number of potential variables, including period, administrative geographic region, and building climate region, a series of statistical tests were conducted using the SPSS 27 software package. The period, geographic region and climate region were analyzed as independent variables, and the correlations between different subgroups were analyzed using Spearman’s rank correlation coefficient. The normality and homogeneity tests were conducted on the corresponding indoor radon concentrations in accordance with the grouping of the independent variables. The data from the different subgroups that met the conditions for normality and homogeneity were analyzed using one-way analysis of variance (ANOVA), while those did not meet the conditions were analyzed using the Kruskal-Wallis test.

The distribution of indoor radon concentrations and the sample size of the survey in China were mapped using ArcGIS software. The data were grouped into three periods: before 2000, 2000–2010, and after 2010. Different colors were used to indicate different radon concentration levels, and the size of distribution points was used to indicate the sample size.

## 3 Results

### 3.1 Database

A total of 37,886 indoor radon concentration data points from 92 papers were collected across three phases of before 2000, 2000–2010 and after 2010, and the summarized results are shown in Table 1.

**Table 1:** The descriptive statistical results of the indoor radon in China.

Period	*N*	Concentration (Bq/m^3^)			Spearman (*p*)	Kruskal-Wallis
		AM	SWM	Range		
Before 2000	17,940	33.2	29.4	0.4 ~ 596	0.494 (0.001)	0.001
2000–2010	10,692	43.3	44.7	1.9 ~ 1,004		
After 2010	9,254	60.8	57.6	1.9 ~ 558		

*N, number of indoor radon survey samples; AM, arithmetic mean; SWM, sample weighted mean.*

Before 2000, the majority of indoor radon concentration data in China were sourced from 37 papers provided by the Ministry of Health, the Ministry of Environmental Protection and other departments. These sources collectively contributed 17,940 data points from 29 provinces. In 2000–2010, the data primarily came from indoor radon surveys organized by the Ministry of Health, the China National Nuclear Corporation and various universities, with a total of 24 studies contributing 10,692 data points from 29 provinces. After 2010, a total of 9,254 indoor radon concentration data points from 31 papers were collected across 21 provinces by universities, colleges, and other departments.

The statistical analysis of the data indicated that there was a significant difference in the mean value of indoor radon concentration across different periods (*p* < 0.001). Additionally, there was a positive correlation between indoor radon concentration in the country and different periods (*p* < 0.001), suggesting that indoor radon concentration tends to increase over time. However, it is important to note that the sample size of the survey has been decreasing, as shown in Table 1.

### 3.2 Measurement methods analysis

The measurement methods employed in indoor radon concentration surveys at various periods in China was shown in Table 2. These measurement methods were classified into three categories based on sampling time, including instantaneous measurements, short-term measurements and long-term measurements. Instantaneous measurements are characterized by the shortest sampling time, which is approximately a few hours. In contrast, short-term measurements, which cover less than 3 months, require a longer sampling duration, while long-term measurements are the most time-consuming and consequently the most reliable.

**Table 2:** The indoor radon measurement methods at different periods in China.

Period		IM/Ratio	STM/Ratio	LTM/Ratio	City
Before 2000	1983–1990	41 (84%)	2 (4%)	6 (12%)	49
	1991–1999	2 (6%)	3 (10%)	26 (84%)	31
2000–2010		2 (3%)	13 (19%)	54 (78%)	69
After 2010		0 (0)	13 (43%)	17 (57%)	30

IM, instantaneous measurement; STM, short-term measurement; LTM, long-term measurement; City, number of cities covered by the surveys in each period.

Before 2000, 54% of indoor radon surveys used instantaneous measurements (such as scintillation flask method, two-Filter Method, and balloon method), 6% used short-term measurements (e.g., activated charcoal detectors, continuous radon monitors), and 40% used long-term measurements (e.g., Alpha-track Detector). However, it was worth noting that most of the indoor radon concentration data in 1983–1990 were measured using instantaneous measurement, while in 1991–1999, the long-term accumulation measurement, which is the Alpha-track Detector method, was the main method for indoor radon concentration measurements. For the indoor radon surveys in the 1980s, due to the limitations of the equipment conditions and measurement methods at that time, as well as the lack of a uniform sampling method, the results of the surveys were often inaccurate in reflecting the average level of radon concentration (20). However, to ensure the accuracy and comparability of the results, three inter-laboratory intercomparisons on grab radon sampling-measurements were organized by the Ministry of Health during the course of survey. At the same time, Ren (3) and Pan (23) also analyzed this issue and considered the national indoor radon concentration levels during this period were of value. Subsequently, in 2000–2010, only two cities were measured using the two-filter method for instantaneous measurements. After 2010, instantaneous measurements were replaced by short-term measurements such as various continuous radon monitors like AlphaGUARD and RAD7, as well as long-term accumulation, such as Alpha-track Detector.

### 3.3 Sampling distribution

**Figure 2:**
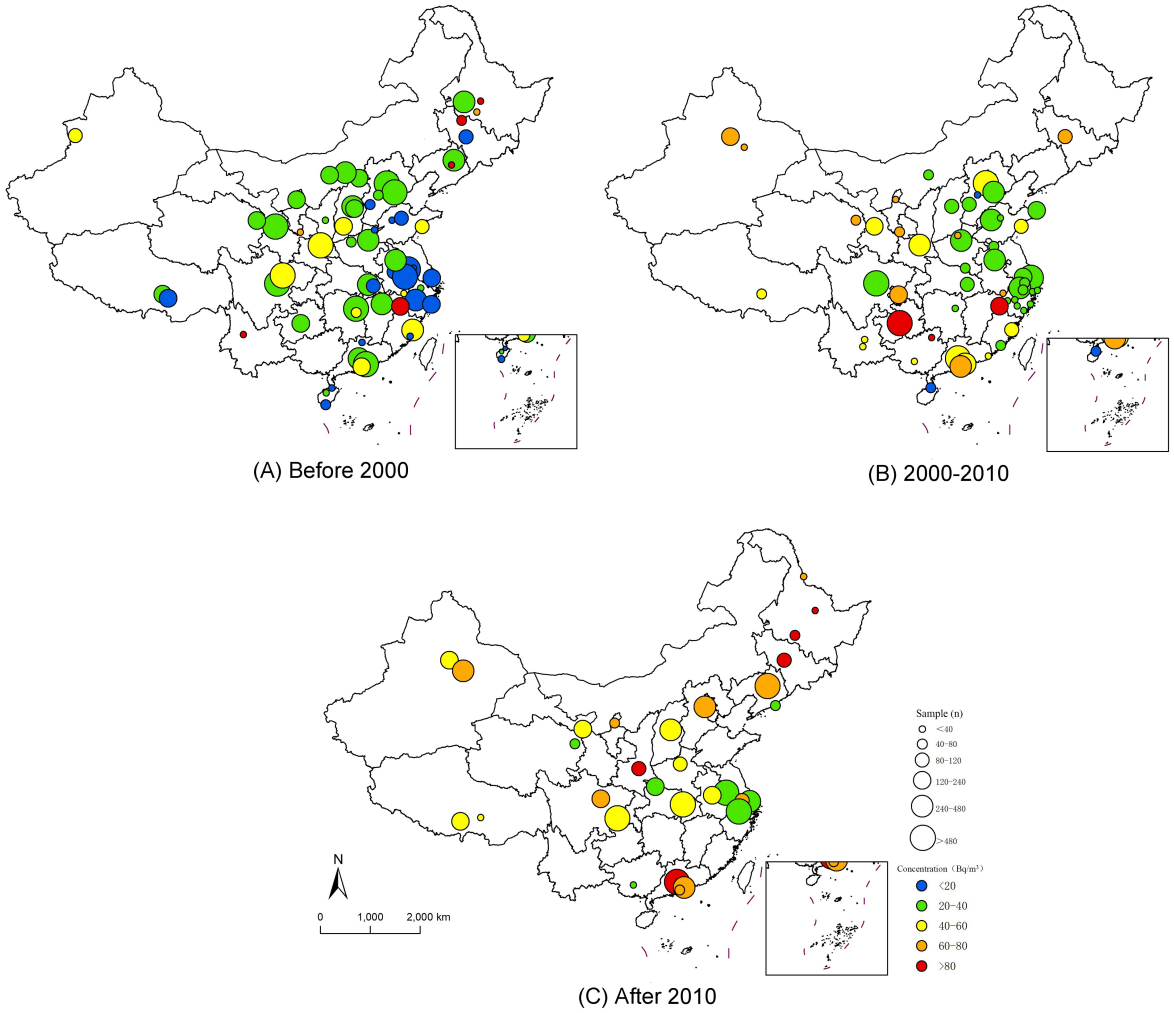
The sampling distribution of indoor radon survey in China.

In order to visualize the distribution of radon concentration and sample size in each investigation, the distribution map of indoor radon concentration and sample size in China has been created based on the data collected in this study, as shown in Figure 2. It can be clearly seen that before 2000, the indoor radon concentration in most of the cities did not exceed 40 Bq/m^3^. However, in 2000–2010, there was a significant increase in the number of cities with indoor radon concentration of more than 40 Bq/m^3^. After 2010, the number of cities with radon concentration below 40 Bq/m^3^ decreased significantly, and the number of cities with average concentration of 80 Bq/m^3^ also increased. For the distribution of sample sizes, it was evident in all three periods that most of the samples were distributed in the eastern part of the country, which may be related to economic development factors and population density. Compared with the period before 2000, the individual sample size and the number of cities in the indoor radon surveys during the other two periods have decreased. Meanwhile, most of the indoor radon surveys after 2010 were limited to the provincial and municipal levels, with no national survey on indoor radon.

### 3.4 Levels and trends of different regions

#### 3.4.1 Administrative region

**Figure 3:**
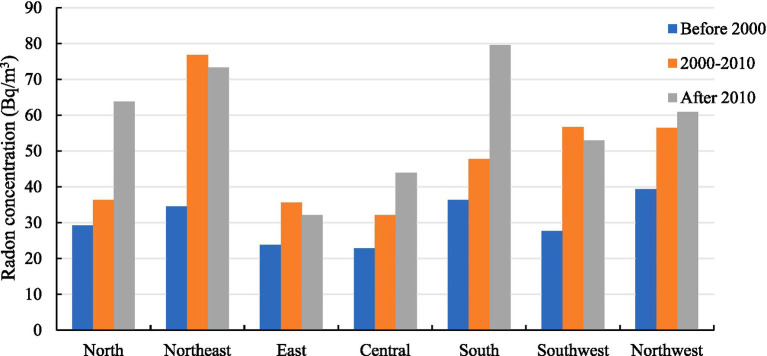
The trends of indoor radon concentration in different administrative regions in China.

China is a vast country with complex topography and significant variations in climate across regions, making regional analyses of indoor radon concentration necessary. Administrative geographic regions represent a comprehensive consideration of political, economic, ethnic, geographical conditions, population distribution, historical tradition and other factors for regional division. Therefore, China was divided into seven administrative regions: North China, Northeast China, East China, Central China, South China, Southwest China, and Northwest China (indoor radon concentration data of Hong Kong, Macao and Taiwan were not collected), and the weighted average indoor radon concentrations in these regions over different periods were summarized in Figure 3. The results of statistical analysis indicated that there were significant differences in indoor radon concentrations between regions in different periods (*p* < 0.05). However, in the correlation analysis, only the indoor radon concentrations in 2000–2010 were correlated with the regions (Spearman correlation coefficient = 0.429, *p* < 0.001).

#### 3.4.2 Climate regions

**Figure 4:**
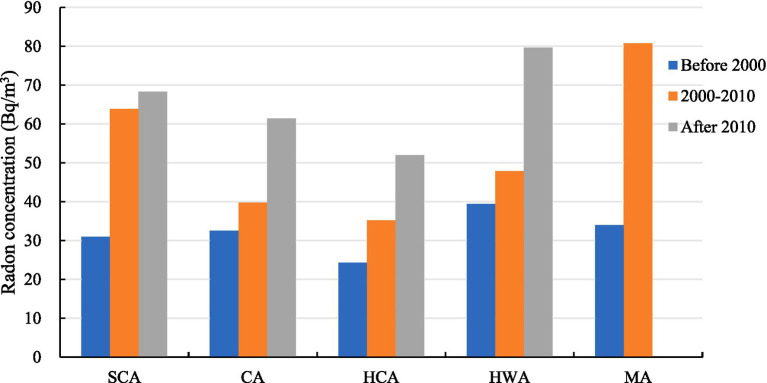
The trends of indoor radon concentration in different climate regions in China.

The building climate region is the climate division of this study, which refers to the Uniform Standard for Design of Civil Buildings (24) and takes into account the differences in building energy-saving design under different climatic conditions in different regions. China was divided into five major climate areas, including severe cold area (SCA), cold area (CA), hot-summer and cold-winter area (HCA), hot-summer and warm-winter area (HWA), and mild area (MA). The indoor radon concentrations in different climate regions in China are shown in Figure 4, and there is no survey data in the mild region after 2010. The indoor radon concentrations in each climate region showed an increasing trend across three periods. The results of statistical analysis showed that the differences in indoor radon concentrations during the two periods, 2000–2010 and after 2010, were significant (*p* < 0.05), but the differences in indoor radon concentrations between climate regions before 2000 were not significant (*p* = 0.15). Meanwhile, the correlation between climate areas and indoor radon concentration was not significant (*p* = 0.206), which was similar to the results of Su (25).

### 3.5 Indoor radon concentration worldwide

A total of 92 data points of national average indoor radon concentration surveys from 63 countries between 1980 and 2018 were collected, and all of them were averaged as arithmetic means. The data were divided into three periods, before 2000, 2000–2010 and after 2010, and the world average indoor radon concentration during the three periods was calculated, shown in Table 3. They were compared with the average indoor radon concentration in China.

**Table 3:** The descriptive statistical results of indoor radon in the world.

Period	Country (*n*)	Concentration (Bq/m^3^)		Reference
		AM	Range	
Before 2000	43	56.5	7 ~ 184	(2, 49, 50)
2000–2010	33	67.9	7.7 ~ 200	(1, 17, 51–61)
After 2010	16	81	41 ~ 189	(15, 27, 62–73)

**Figure 5:**
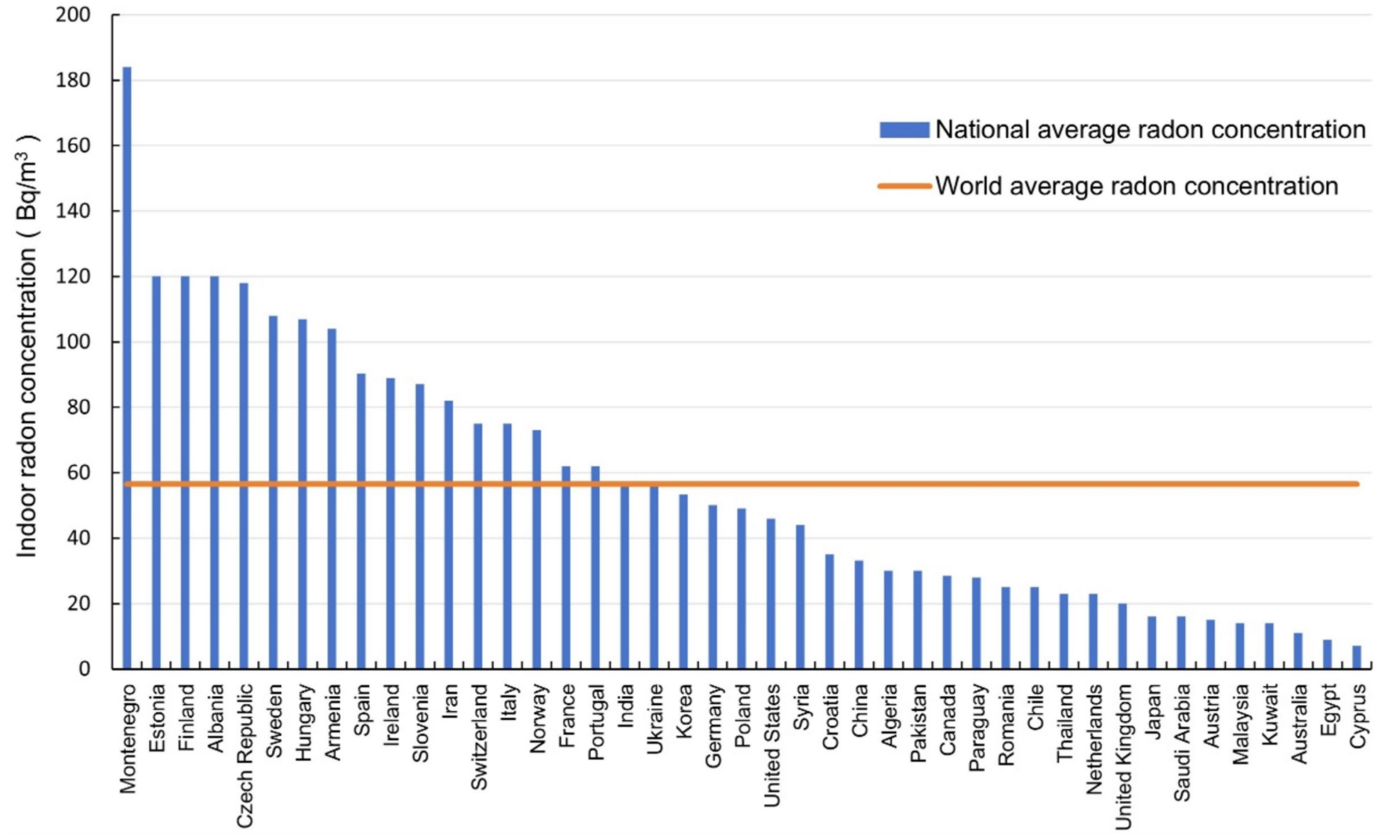
The average indoor radon concentration in different countries before 2000.

**Figure 6:**
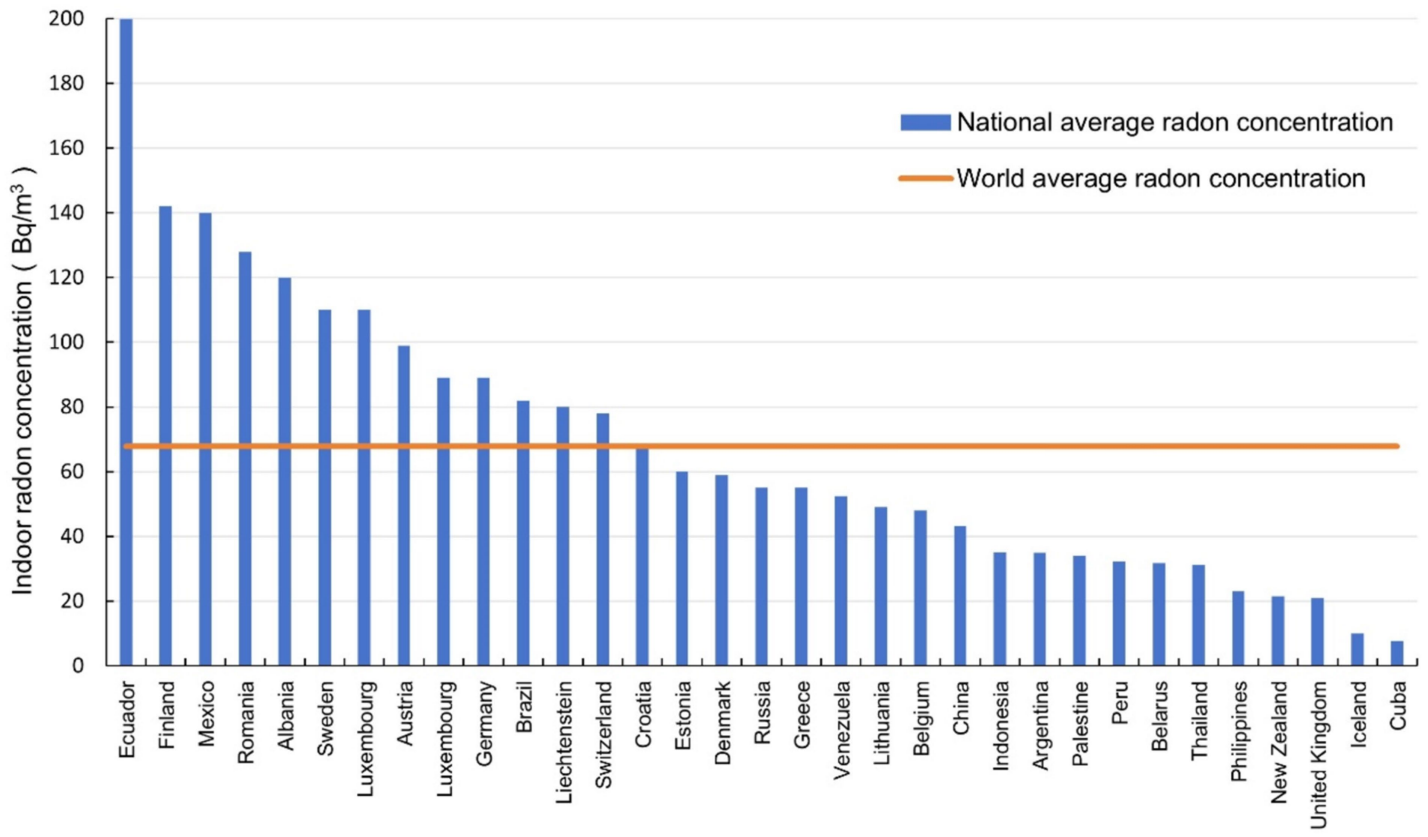
The average indoor radon concentration in different countries during 2000–2010.

**Figure 7:**
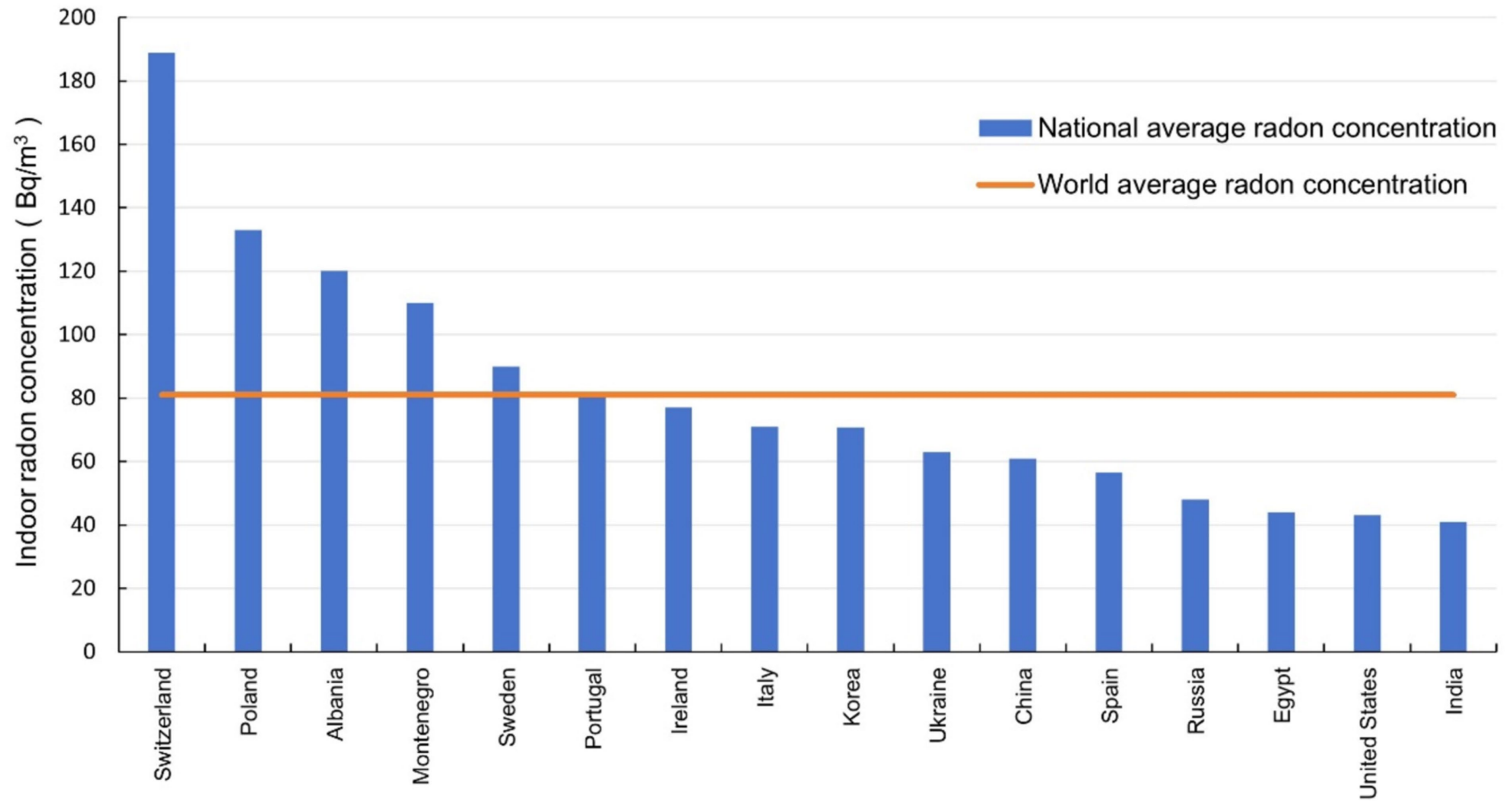
The average indoor radon concentration in different countries after 2010.

The average indoor radon concentrations in different countries in three periods were shown in Figures 5–7 respectively. It can be seen that the indoor radon concentrations in China in three periods (25.6 Bq/m^3^, 43.3 Bq/m^3^, and 60.8 Bq/m^3^) were in the middle of the world, lower than the world average value. Comparing the data of countries in the three periods, it is evident that the average indoor radon concentration in the world has been increasing.

**Figure 8:**
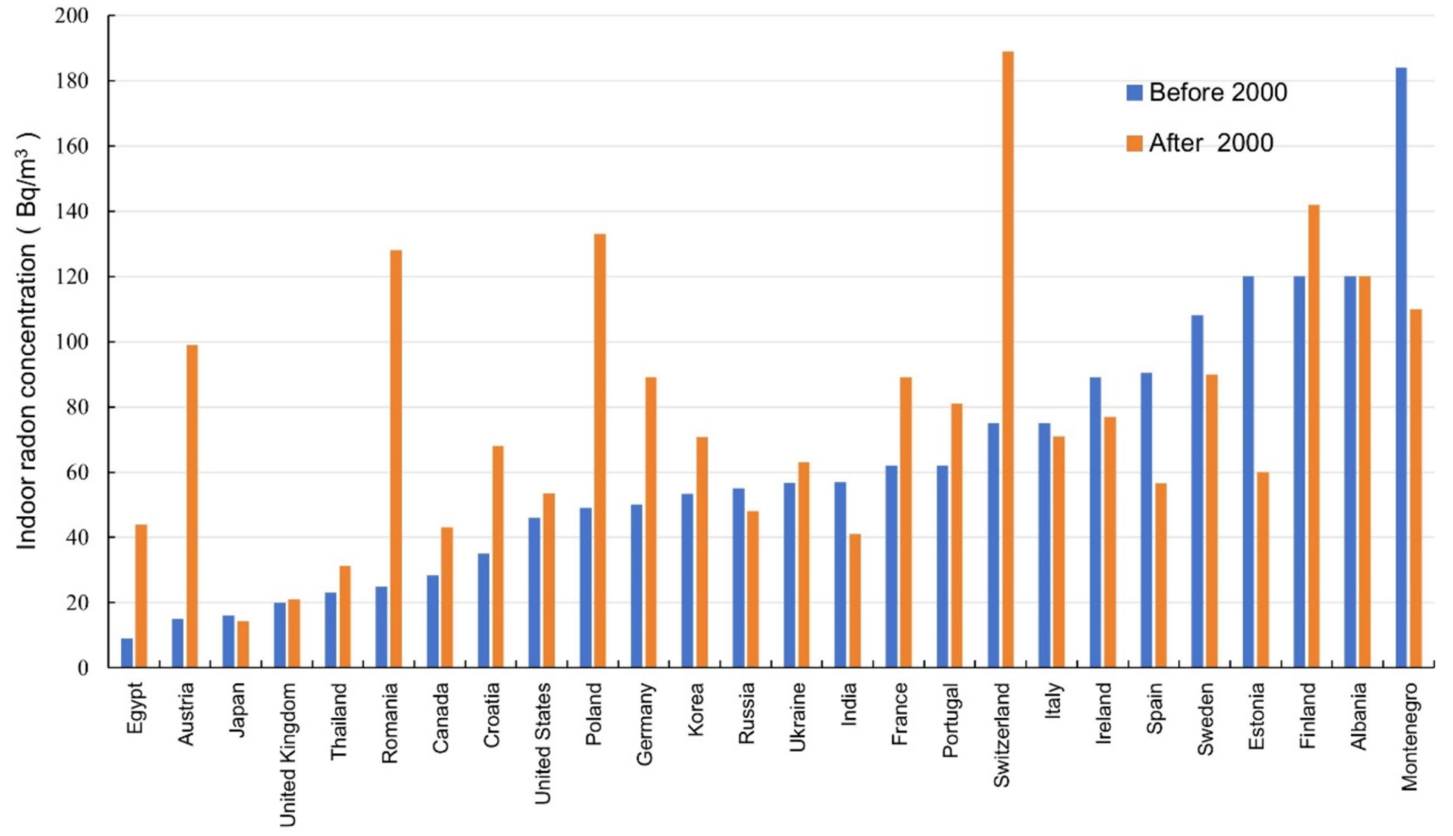
Comparison of average indoor radon concentrations in 26 countries before and after 2000.

The 26 countries with indoor radon concentration surveys before and after 2000 were collected in the database, as shown in Figure 8. Sixteen of them showed an increasing trend of indoor radon concentration before and after 2000, with an increase of 5% to 560%. Nine countries showed a decreasing trend in indoor radon concentration, with a decrease of 5% to 50%. Albania, which is located in the southeastern part of Europe, did not show any significant change. The indoor radon concentration in most countries was in increasing trend and the increase was large, while the indoor radon concentration in some countries was decreasing and the decrease was small.

## 4 Discussion

This study presented a summary of data from indoor radon surveys conducted in China and other countries between the 1980s and 2023. The levels and trends of indoor radon concentrations were analyzed with respect to the different periods, measurement methods and regions.

The results showed that the weighted and arithmetic mean radon concentrations in China were 29.4 Bq/m^3^ and 33.2 Bq/m^3^ before 2000, 44.7 Bq/m^3^ and 43.3 Bq/m^3^ in 2000–2010, 57.6 Bq/m^3^ and 60.8 Bq/m^3^ after 2010, respectively. It is obvious that the indoor radon concentration in China showed an increasing trend, which was basically consistent with the conclusion of the study of Su (25) and Yao (26).

A number of factors could contribute to elevated indoor radon concentrations. These include indoor and outdoor environments (27), geologic radon potential (28, 29), soil radium content and surface precipitation rates (30), climate and season (31–34), building type, building structure, and age of the building (27). In addition, the most widely reported factors are building materials and ventilation (35–39). In recent years, China has actively promoted the use of new building materials in order to meet the requirements of energy conservation and environmental protection. The autoclaved aerated concrete has emerged as a highly suitable alternative to traditional solid clay bricks. However, the porosity of aerated concrete was high, and the radon exhalation rate of building materials was significantly higher than that of fly ash bricks, coal gangue bricks, and clay bricks (35), resulting in elevated indoor radon concentrations in new dwellings utilizing aerated concrete blocks as building wall materials compared to those utilizing brick-concrete. In terms of ventilation, the indoor radon concentration in buildings with closed doors and windows was considerably higher than that in poorly ventilated and normally ventilated buildings (25). Furthermore, the energy-saving design of buildings results in increased air tightness and a decreased rate of air exchange, which ultimately led to the accumulation of radon concentration indoors. For example, in 2020, Wu (40) measured that the average air exchange rate of 21 houses in five cities (0.19 h^−1^) was significantly lower than Ren’s (41) measurements of typical buildings in Beijing in the 1980s (0.50 h^−1^). Meanwhile, a study by Vasilyev (39) of Russia showed that radon concentrations in modern energy-efficient buildings are two times higher than in old buildings, and that the low air exchange rate of energy-efficient buildings is the main factor contributing to their elevated indoor radon concentrations. Arvela’s study in Finland (42) showed that the indoor radon concentration in new energy-efficient buildings with an air exchange rate of 0.6 h^−1^ is two times higher than in old buildings with an air exchange rate of 4 h^−1^.

There were significant differences in indoor radon concentrations across different administrative geographic regions and building climate regions. The indoor radon concentration in the Northwest China, South China and Northeast China was higher before 2000, the indoor radon concentration in the Northeast China, Southwest China and Northwest China was higher from 2000 to 2010, and the indoor radon concentration in the South China, Northeast China and North China was higher after 2010. It can be observed that the indoor radon concentration in the three northern regions is consistently higher across all three time periods in China. This may be attributed to the region’s habit of opening windows and ventilating less during the winter months due to the severe cold climate (43). Additionally, the majority of indoor radon samples from South China originate from Guangzhou. For instance, after 2010, there were 2,224 samples from South China, of which 1,796 were from Guangzhou (44). The average indoor radon concentration in this region is notably high, reaching 84.2 Bq/m^3^. This may be attributed to the elevated radioactivity levels of concrete rubble and fly ash utilized in construction in Guangzhou, which contribute to the radioactivity of formed concrete being considerably higher than that observed in China and globally (45). The indoor radon concentrations in SCA, CA, HCA, HWA, and HCA all demonstrated an increasing trend across the three periods. Among them, the indoor radon concentration in SCA and CA is higher compared to other areas. A systematic review of indoor radon in China from 2000 to 2020 also found that the indoor radon concentrations in SCA and CA in winter are on the high side (25). The indoor radon concentration in MA was higher than other climate areas both before 2000 and in 2000–2010, which may be related to the high soil ^226^Ra content and radon surface precipitation rates in this climate area (30). Soil gas is a very stable radon source and is capable of providing steady non depleting ^222^Rn concentration over a long period of operation (46, 47). Mainly entering through cracks or holes in foundations and concrete floors as a consequence of pressure differential between buildings and the surrounding soil, radon gas concentrations can reach high indoor levels (48), especially in low-rise buildings where the foundation is closer to the soil.

The results of this paper presented in this paper indicated that the global average indoor radon concentration was increasing, with the arithmetic mean radon concentrations across the three periods being 56.5 Bq/m^3^, 67.9 Bq/m^3^ and 81 Bq/m^3^, respectively. The indoor radon concentrations in 9 countries showed a decreasing trend when comparing before and after 2000, while 16 countries’ indoor radon concentration showed an increasing trend, indicating that the increase of indoor radon concentration in China was not an isolated case, and the issue of indoor radon pollution still required further attention.

## 5 Limitations

The samples varied in quality in this study. The indoor radon concentration data of different sample sizes were obtained by different organizations and investigators using different measurement methods.

The distribution of samples was uneven. Firstly, most of the indoor radon data came from homes, with less from offices and public places. Secondly, the samples of each province may only come from one city, which means that indoor radon in other areas was neglected. Finally, the samples for different regions varied greatly. Some regions lack data for certain periods, which may lead to bias in the results.

Sample weighting was inherently limiting. Different sample sizes would affect the average radon concentration. The sample-weighted approach was used to calculate the average indoor radon concentration at the provincial, regional and national levels, which could reduce the effect of small sample size surveys and make the obtained average radon concentration values more biased towards surveys with large sample sizes.

Therefore, it is essential to apply uniformly indoor radon measurement methods (e.g., alpha-track detectors) which were calibrated to ensure the effectiveness of surveys in future. At the same time, quality assurance and control measures must be implemented to guarantee the accuracy and reliability of the data. Samples should be collected from as many districts and diverse types of buildings (e.g., dwellings, offices, and public places) as possible across various provinces and regions, in order to accurately reflect the distribution of indoor radon concentrations nationwide. Additionally, the distribution of the population across different areas should be considered, with sample data weighted according to population proportions, to obtain a more accurate regional and national average radon concentration.

## References

[1] United Nations. Effects of ionizing radiation. Volume II: Sources-to-effects assessment for radon in homes and workplaces. United Nations Scientifc committee on the effects of atomic radiation, 2006. Report to the general assembly, with scientifc annexes. United Nations, New York: United Nations sales publications E.09.IX.5 (2006).

[2] United Nations. Sources and effects of lonizing radiation. Volume I: Sources; volume I: Effects. United Nations Scientifc committee on the Effectsof atomic radiation, 2000 report to the general assembly, with scientifc annexes. United Nations, New York: United Nations sales publications E.00.1X.3 and E.00.IX.4 (2000).

[3] Ren T. Source, level and control of indoor radon. Radiat Prot. (2001) 21:291–9. doi: 10.3321/j.issn:1000-8187.2001.05.005

[4] Biological Effects of Ionizing Radiation VI Report. Health effects of exposure to indoor radon. Washington D.C.: BEIR, National Academy Press (1999).

[5] Darby S Hill D Auvinen A Barros-Dios JM Baysson H Bochicchio F. Radon in homes and risk of lung cancer: collaborative analysis of individual data from 13 European case-control studies. BMJ. (2005) 330:223. doi: 10.1136/bmj.38308.477650.63, PMID: 15613366 PMC546066

[6] Darby S Hill D Deo H Auvinen A Barros-Dios JM Baysson H. Residential radon and lung cancer--detailed results of a collaborative analysis of individual data on 7148 persons with lung cancer and 14,208 persons without lung cancer from 13 epidemiologic studies in Europe. Scand J Work Environ Health. (2006) 32:1–83. Available at: https://www.ncbi.nlm.nih.gov/pubmed/16538937 16538937

[7] Grosche B Kreuzer M Kreisheimer M Schnelzer M Tschense A. Lung cancer risk among German male uranium miners: a cohort study, 1946–1998. Br J Cancer. (2006) 95:1280–7. doi: 10.1038/sj.bjc.6603403, PMID: 17043686 PMC2360564

[8] Krewski D Lubin JH Zielinski JM Alavanja M Catalan VS Field RW. Residential radon and risk of lung cancer: a combined analysis of 7 north American case-control studies. Epidemiology. (2005) 16:137–45. doi: 10.1097/01.ede.0000152522.80261.e3, PMID: 15703527

[9] Krewski D Lubin JH Zielinski JM Alavanja M Catalan VS Field RW. A combined analysis of north American case-control studies of residential radon and lung cancer. J Toxicol Environ Health A. (2006) 69:533–97. doi: 10.1080/15287390500260945, PMID: 16608828

[10] Lubin JH Wang ZY Boice JD Jr Xu ZY Blot WJ De Wang L. Risk of lung cancer and residential radon in China: pooled results of two studies. Int J Cancer. (2004) 109:132–7. doi: 10.1002/ijc.11683, PMID: 14735479

[11] Tomasek L Rogel A Tirmarche M Mitton N Laurier D. Lung cancer in French and Czech uranium miners: radon-associated risk at low exposure rates and modifying effects of time since exposure and age at exposure. Radiat Res. (2008) 169:125–37. doi: 10.1667/rr0848.1 18220460

[12] WHO Guidelines Approved by the Guidelines Review Committee. WHO Handbook on Indoor Radon: A Public Health Perspective. Geneva: World Health Organization (2009). 23762967

[13] Cincinelli A Martellini T. Indoor air quality and health. Int J Environ Res Public Health. (2017) 14:1286. doi: 10.3390/ijerph14111286, PMID: 29068361 PMC5707925

[14] Wanner H Kuhn M. Indoor air pollution by building materials. Environ Int. (1986) 12:311–5. doi: 10.1016/0160-4120(86)90044-9

[15] Dowdall A Murphy P Pollard D Fenton D. Update of Ireland's national average indoor radon concentration – application of a new survey protocol. J Environ Radioact. (2017) 169–170:1–8. doi: 10.1016/j.jenvrad.2016.11.034 28027495

[16] Savković ME Udovičić V Maletić D Pantelić G Ujić P Čeliković I. Results of the first national indoor radon survey performed in Serbia. J Radiol Prot. (2020) 40:N22–30. doi: 10.1088/1361-6498/ab749e, PMID: 32040947

[17] Suzuki G Yamaguchi I Ogata H Sugiyama H Yonehara H Kasagi F. A nation-wide survey on indoor radon from 2007 to 2010 in Japan. J Radiat Res. (2010) 51:683–9. doi: 10.1269/jrr.10083, PMID: 20940519

[18] Cheng J Guo Q Ren T. Radon levels in China. J Nucl Sci Technol. (2002) 39:695–9. doi: 10.1080/18811248.2002.9715251

[19] Jinhua S. Survey of concentrations of radon and α potential energy of radon daughter productes in air in some regions of China (1983–1990). National Environmental Natural Radioactivity Level Survey Summary Report Compilation Group. Radiat Prot. (1992) 12:164–71. Available at: https://d.wanfangdata.com.cn/periodical/ChlQZXJpb2RpY2FsQ0hJTmV3UzIwMjQwNzA0Eg5RSzAwMDAwMDQ3NTgwNBoIcXlhYmVyNG8%3D

[20] Shang B He Q Wang Z Zhu C. Studies of indoor action level of radon in China. Chin J Radiol Med Prot. (2003) 23:462–5. doi: 10.3760/cma.j.issn.0254-5098.2003.06.032

[21] Wang C Pan Z Liu S Yang M Shang B Zhuo W. A survey and study of radon concentration levels in living rooms in selected cities in China. Radiat Prot. (2014). 34:65–73. doi: 10.3969/j.issn.1000-8187.2014.02.001

[22] Jing J Lu X Li C Fu L Shi L Qiang L. Study indoor Eadon concentration levels and human radiation exposure in Xi'an City. Chin J Radiol Health. (2015) 24:167–9.

[23] Pan Z. Exposure resulted from radon and its decay products in air in China. Radiat Prot. (2003) 23:129–37,83. doi: 10.3321/j.issn:1000-8187.2003.03.001

[24] Ministry of Housing and Urban-Rural Development of the People's Republic of China. Uniform standard for Design of Civil Buildings: GB50352-2019. (2019).

[25] Su C Pan M Zhang Y Kan H Zhao Z Deng F. Indoor exposure levels of radon in dwellings, schools, and offices in China from 2000 to 2020: a systematic review. Indoor Air. (2022) 32:e12920. doi: 10.1111/ina.12920, PMID: 34432341

[26] Yao Y Chen B Zhuo W. Reanalysis of residential radon surveys in China from 1980 to 2019. Sci Total Environ. (2021) 757:143767. doi: 10.1016/j.scitotenv.2020.143767, PMID: 33234270

[27] Kropat G Bochud F Jaboyedoff M Laedermann J-P Murith C Palacios M. Major influencing factors of indoor radon concentrations in Switzerland. J Environ Radioact. (2014) 129:7–22. doi: 10.1016/j.jenvrad.2013.11.010, PMID: 24333637

[28] Demoury C Ielsch G Hemon D Laurent O Laurier D Clavel J. A statistical evaluation of the influence of housing characteristics and geogenic radon potential on indoor radon concentrations in France. J Environ Radioact. (2013) 126:216–25. doi: 10.1016/j.jenvrad.2013.08.006, PMID: 24056050

[29] Sundal AV Henriksen H Soldal O Strand T. The influence of geological factors on indoor radon concentrations in Norway. Sci Total Environ. (2004) 328:41–53. doi: 10.1016/j.scitotenv.2004.02.011, PMID: 15207572

[30] Zhuo W Chen B Li D Liu H. Reconstruction of database on natural radionuclide contents in soil in China. J Nucl Sci Technol. (2008) 45:180–4. doi: 10.1080/00223131.2008.10876003

[31] Bossew P Lettner H. Investigations on indoor radon in Austria, part 1: seasonality of indoor radon concentration. J Environ Radioact. (2007) 98:329–45. doi: 10.1016/j.jenvrad.2007.06.006, PMID: 17707559

[32] Espinosa G Gammage R. An indoor radon survey in three different climate regions in Mexico, and the influence of climate in the obtained values. J Environ Prot. (2011) 2:1143–8. doi: 10.4236/jep.2011.29133

[33] Magalhães M Amaral E Sachett I Rochedo E. Radon-222 in Brazil: an outline of indoor and outdoor measurements. J Environ Radioact. (2003) 67:131–43. doi: 10.1016/S0265-931X(02)00175-3, PMID: 12660045

[34] Vaupotič J Kobal I Planinić J. Long-term radon investigation in four selected kindergartens in different geological and climate regions of Slovenia. J Radioanal Nucl Chem. (1998) 238:61–6. doi: 10.1007/BF02385356

[35] Ge L Chen Y Li F Xu J Li H. Measurement of 226Ra concentration and radon precipitation rate of new wall materials mixed with industrial waste residues. Chin J Radiol Med Prot. (2008) 28:404–5. doi: 10.3760/cma.j.issn.0254-5098.2008.04.032

[36] Ashok GV Nagaiah N Shiva Prasad NG. Indoor radon concentration and its possible dependence on ventilation rate and flooring type. Radiat Prot Dosim. (2012) 148:92–100. doi: 10.1093/rpd/ncq590, PMID: 21335628

[37] Chauhan R Kumar A Chauhan N Joshi M Aggarwal P Sahoo B. Ventilation effect on indoor radon–thoron levels in dwellings and correlation with soil exhalation rates. Indoor Built Environ. (2016) 25:203–12. doi: 10.1177/1420326X14542887

[38] Lembrechts J Janssen M Stoop P. Ventilation and radon transport in Dutch dwellings: computer modelling and field measurements. Sci Total Environ. (2001) 272:73–8. doi: 10.1016/s0048-9697(01)00667-2, PMID: 11379940

[39] Vasilyev AV Yarmoshenko IV Zhukovsky MV. Low air exchange rate causes high indoor radon concentration in energy-efficient buildings. Radiat Prot Dosim. (2015) 164:601–5. doi: 10.1093/rpd/ncv319, PMID: 25977350

[40] Wu Y Zhang Q Song Y Shang B Cui H. Impact of energy-saving design of residential buildings on both indoor radon concentration and air exchange rate in severe-cold areas and cold areas. Chin J Radiol Med Prot. (2020) 40:945–50. doi: 10.3760/cma.j.issn.0254-5098.2020.12.009

[41] Ren T. Environmental radiation measurement and evaluation. Beijing: Atomic Energy Publishing (2005).

[42] Arvela H Holmgren O Reisbacka H Vinha J. Review of low-energy construction, air tightness, ventilation strategies and indoor radon: results from Finnish houses and apartments. Radiat Prot Dosim. (2014) 162:351–63. doi: 10.1093/rpd/nct278, PMID: 24243314

[43] Wu Y Song Y Zhang Q Shang B Cui H Hou C. Indoor radon concentration and its changing trend in northeastern China. Chin J Radiol Health. (2023) 32:115–8,30. doi: 10.13491/j.issn.1004-714X.2023.02.005

[44] Mei A Liu Z Pan W. Survey on radon concentration in recently built residences of Guangzhou. Guangzhou Archit. (2016) 2:14–7. doi: 10.3969/j.issn.1671-2439.2016.02.004

[45] Mei A Pan W. Evaluation of radioactivity level of concrete raw materials in an administrative district of Guangzhou. Chem Eng. (2014) 1:88–90. doi: 10.3969/j.issn.1007-0389.2014.01.036

[46] Karunakara N Shetty T Sahoo BK Kumara KS Sapra BK Mayya YS. An innovative technique of harvesting soil gas as a highly efficient source of (222)Rn for calibration applications in a walk-in type chamber: part-1. Sci Rep. (2020) 10:16547. doi: 10.1038/s41598-020-73320-9, PMID: 33024139 PMC7538554

[47] Shetty T Mayya YS Kumara KS Sahoo BK Sapra BK Karunakara N. A periodic pumping technique of soil gas for (222)Rn stabilization in large calibration chambers: part 2-theoretical formulation and experimental validation. Sci Rep. (2020) 10:16548. doi: 10.1038/s41598-020-71872-4, PMID: 33024133 PMC7538436

[48] Borgoni R De Francesco D De Bartolo D Tzavidis N. Hierarchical modeling of indoor radon concentration: how much do geology and building factors matter? J Environ Radioact. (2014) 138:227–37. doi: 10.1016/j.jenvrad.2014.08.022, PMID: 25261869

[49] Létourneau EG Krewski D Zielinski JM McGregor RG. Cost effectiveness of radon mitigation in Canada. Radiat Prot Dosim. (1992) 45:593–8. doi: 10.1093/oxfordjournals.rpd.a081611

[50] Pavlenko T Los I Aksenov N. Indoor 222Rn levels and irradiation doses in the territory of the Ukraine. Radiat Meas. (1996) 26:585–91. doi: 10.1016/1350-4487(95)00307-X

[51] Leenhouts H Brugmans M. Calculation of the 1995 lung cancer incidence in the Netherlands and Sweden caused by smoking and radon: risk implications for radon. Radiat Environ Biophys. (2001) 40:11–21. doi: 10.1007/s004110000084, PMID: 11357706

[52] Pahapill L Rulkov A Rajamäe R Åkerblom G. Radon in Estonian dwellings-results from a National Radon Survey. (No. SSI--2003-16). Swedish Radiation Protection Authority. (2003).

[53] Billon S Morin A Caer S Baysson H Gambard J Backe J. French population exposure to radon, terrestrial gamma and cosmic rays. Radiat Prot Dosim. (2005) 113:314–20. doi: 10.1093/rpd/nch463, PMID: 15713740

[54] Friedmann H. Final results of the Austrian radon project. Health Phys. (2005) 89:339–48. doi: 10.1097/01.HP.0000167228.18113.27, PMID: 16155455

[55] Wanabongse P Tokonami S Bovornkitti S eds. Current studies on radon gas in Thailand. Elsevier Science: International Congress Series Elsevier Science (2005) 1276:208–209.

[56] Catelinois O Rogel A Laurier D Billon S Hemon D Verger P. Lung cancer attributable to indoor radon exposure in France: impact of the risk models and uncertainty analysis. Environ Health Perspect. (2006) 114:1361–6. doi: 10.1289/ehp.9070, PMID: 16966089 PMC1570096

[57] Zeeb H. World Health Organization. International radon project. Survey on radon guidelines, programmes and activities. Final report. (No. WHO/HSE/RAD/07.01). World Health Organization. (2007).

[58] Menzler S Piller G Gruson M Rosario AS Wichmann H-E Kreienbrock L. Population attributable fraction for lung cancer due to residential radon in Switzerland and Germany. Health Phys. (2008) 95:179–89. doi: 10.1097/01.HP.0000309769.55126.03, PMID: 18617799

[59] Gray A Read S McGale P Darby S. Lung cancer deaths from indoor radon and the cost effectiveness and potential of policies to reduce them. BMJ. (2009) 338:a3110. doi: 10.1136/bmj.a3110, PMID: 19129153 PMC2769068

[60] Truta-Popa L-A Dinu A Dicu T Szacsvai K Cosma C Hofmann W. Preliminary lung cancer risk assessment of exposure to radon progeny for Transylvania, Romania. Health Phys. (2010) 99:301–7. doi: 10.1097/HP.0b013e3181c03cde, PMID: 20699690

[61] Valmari T Maekelaeinen I Reisbacka H Arvela H. Radon atlas of Finland 2010; Suomen radonkartasto 2010. (No. STUK-A--245). Radiation and Nuclear Safety Authority STUK (2010).

[62] Axelsson G Andersson EM Barregard L. Lung cancer risk from radon exposure in dwellings in Sweden: how many cases can be prevented if radon levels are lowered? Cancer Causes Control. (2015) 26:541–7. doi: 10.1007/s10552-015-0531-6, PMID: 25677843 PMC4365178

[63] Bochicchio F Antignani S Venoso G Forastiere F. Quantitative evaluation of the lung cancer deaths attributable to residential radon: a simple method and results for all the 21 Italian regions. Radiat Meas. (2013) 50:121–6. doi: 10.1016/j.radmeas.2012.09.011

[64] Fernández CS Poncela LQ Villar AF Merino IF Gutierrez-Villanueva J González SC. Spanish experience on the design of radon surveys based on the use of geogenic information. J Environ Radioact. (2017) 166:390–7. doi: 10.1016/j.jenvrad.2016.07.007, PMID: 27681529

[65] Kumar A Chauhan R Joshi M Aggarwal P. Implications of variability in indoor radon/thoron levels: a study of dwellings in Haryana, India. Environ Earth Sci. (2015) 73:4033–42. doi: 10.1007/s12665-014-3688-5

[66] Park TH Kang DR Park SH Yoon DK Lee CM. Indoor radon concentration in Korea residential environments. Environ Sci Pollut Res. (2018) 25:12678–85. doi: 10.1007/s11356-018-1531-3, PMID: 29468397

[67] Pavlenko T German O Frizyuk M Aksenov N Operchyuk A. The Ukrainian pilot project “stop radon”. Nucl Technol Radiat Prot. (2014) 29:142–8. doi: 10.2298/NTRP1402142P

[68] Peterson E Aker A Kim J Li Y Brand K Copes R. Lung cancer risk from radon in Ontario, Canada: how many lung cancers can we prevent? Cancer Causes Control. (2013) 24:2013–20. doi: 10.1007/s10552-013-0278-x, PMID: 23982909 PMC3824583

[69] Przylibski TA Żebrowski A Karpińska M Kapała J Kozak K Mazur J. Mean annual 222Rn concentration in homes located in different geological regions of Poland–first approach to whole country area. J Environ Radioact. (2011) 102:735–41. doi: 10.1016/j.jenvrad.2011.03.018, PMID: 21555169

[70] Turner MC Krewski D Chen Y Pope CA 3rd Gapstur SM Thun MJ. Radon and nonrespiratory mortality in the American Cancer Society cohort. Am J Epidemiol. (2012) 176:808–14. doi: 10.1093/aje/kws198, PMID: 23045472

[71] Veloso B Nogueira JR Cardoso MF. Lung cancer and indoor radon exposure in the north of Portugal–an ecological study. Cancer Epidemiol. (2012) 36:e26–32. doi: 10.1016/j.canep.2011.10.005, PMID: 22075535

[72] Vukotic P Antovic N Djurovic A Zekic R Svrkota N Andjelic T. Radon survey in Montenegro–a base to set national radon reference and “urgent action” level. J Environ Radioact. (2019) 196:232–9. doi: 10.1016/j.jenvrad.2018.02.009, PMID: 29501265

[73] Yarmoshenko I Malinovsky G Vasilyev A Zhukovsky M. Reconstruction of national distribution of indoor radon concentration in Russia using results of regional indoor radon measurement programs. J Environ Radioact. (2015) 150:99–103. doi: 10.1016/j.jenvrad.2015.08.007, PMID: 26313426

