# Corrigendum: Analysis of indoor radon concentration levels and trends in China

**DOI:** 10.3389/fpubh.2025.1603240

**Published:** 2025-04-14

**Authors:** Bowei Ding, Yunyun Wu, Yanchao Song, Changsong Hou, Bing Shang

**Affiliations:** Key Laboratory of Radiological Protection and Nuclear Emergency, China CDC, National Institute for Radiological Protection Chinese Center for Disease Control and Prevention, Beijing, China

**Keywords:** China, radon, indoor, database, regional analyses

In the published article, there was an error in Figure 2 as published. We have identified a labeling error in Figure 2C of our paper. The current timeline label reads “After 2000” but should be corrected to “After 2010” to accurately reflect the sampling period distribution. The corrected Figure 2 appear below.

**Figure 2:**
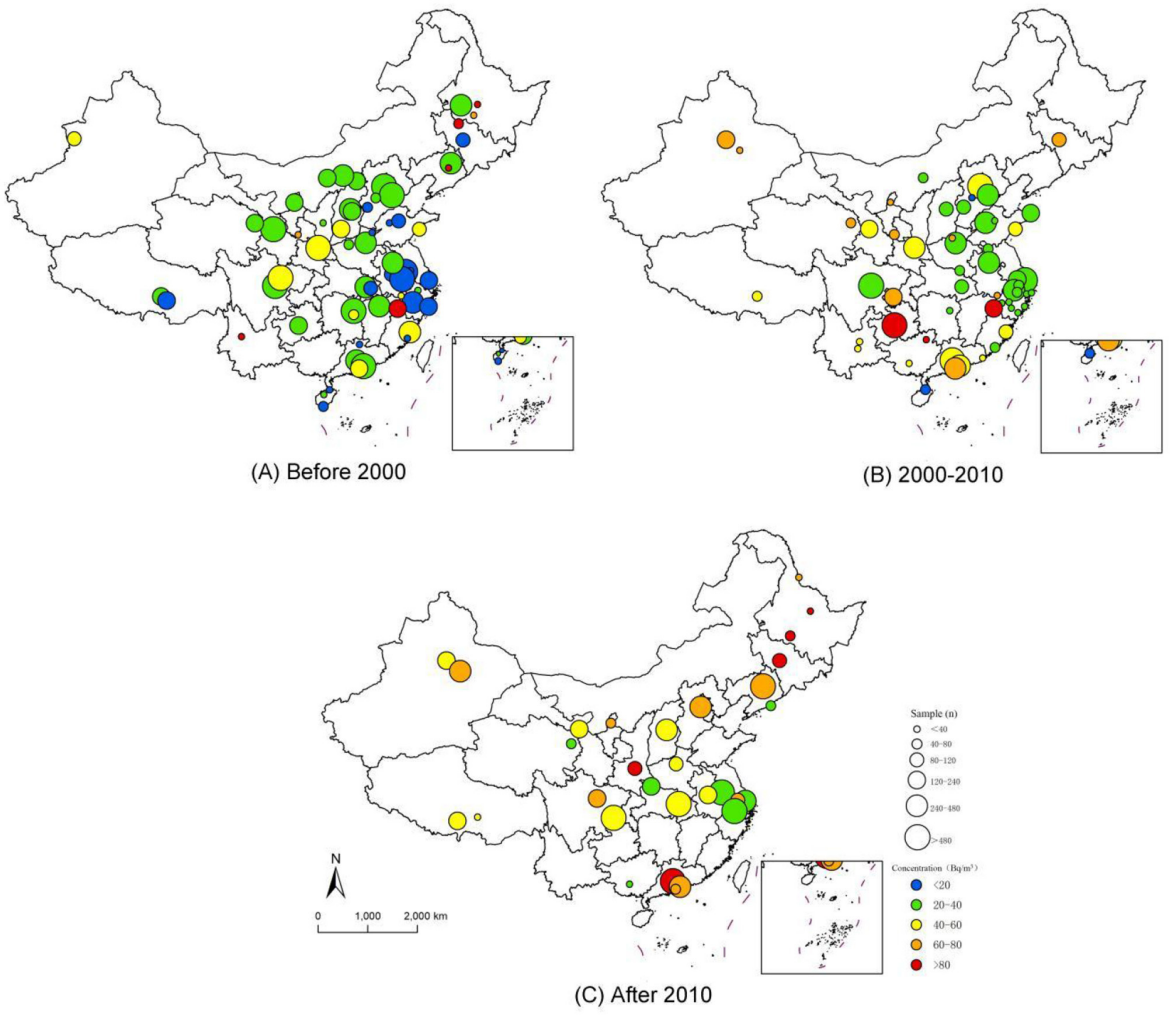
The sampling distribution of indoor radon survey in China.

The authors apologize for this error and state that this does not change the scientific conclusions of the article in any way. The original article has been updated.

